# Secondary production and priming reshape the organic matter composition in marine sediments

**DOI:** 10.1126/sciadv.adm8096

**Published:** 2024-05-17

**Authors:** Qing-Zeng Zhu, Xiuran Yin, Heidi Taubner, Jenny Wendt, Michael W. Friedrich, Marcus Elvert, Kai-Uwe Hinrichs, Jack J. Middelburg

**Affiliations:** ^1^ MARUM Center for Marine Environmental Sciences, University of Bremen, Bremen, Germany.; ^2^ Department of Earth Sciences, Utrecht University, Utrecht, Netherlands.; ^3^ Microbial Ecophysiology Group, Faculty of Biology/Chemistry, University of Bremen, Bremen, Germany.; ^4^ State Key Laboratory of Marine Resource Utilization in South China Sea, Hainan University, Haikou, China.; ^5^ Faculty of Geosciences, University of Bremen, Bremen, Germany.

## Abstract

Organic matter (OM) transformations in marine sediments play a crucial role in the global carbon cycle. However, secondary production and priming have been ignored in marine biogeochemistry. By incubating shelf sediments with various ^13^C-labeled algal substrates for 400 days, we show that ~65% of the lipids and ~20% of the proteins were mineralized by numerically minor heterotrophic bacteria as revealed by RNA stable isotope probing. Up to 11% of carbon from the algal lipids was transformed into the biomass of secondary producers as indicated by ^13^C incorporation in amino acids. This biomass turned over throughout the experiment, corresponding to dynamic microbial shifts. Algal lipid addition accelerated indigenous OM degradation by 2.5 to 6 times. This priming was driven by diverse heterotrophic bacteria and sulfur- and iron-cycling bacteria and, in turn, resulted in extra secondary production, which exceeded that stimulated by added substrates. These interactions between degradation, secondary production, and priming govern the eventual fate of OM in marine sediments.

## Teaser

Interactions between degradation, secondary production, and priming govern the fate of OM in marine sediments.

## INTRODUCTION

Organic matter (OM) in marine sediments is a pivotal component of the global carbon cycle (*1*, *2*). Sedimentary OM predominantly originates from primary production in the sunlit layers of the ocean. The majority of the OM from primary production is consumed in the photic zone or degraded while settling through the water column, with the result that only a few percent eventually reaches the seafloor (*1*–*3*). After deposition, benthic animals or microbes consume and transform OM (*3*). Eventually, the remaining OM becomes resistant to biological degradation, molecularly uncharacterizable, and buried in the deep biosphere for geological times (*3*–*5*).

The conventional view of sedimentary OM processing focuses dominantly on degradation (*6*, *7*). However, the organisms consuming OM synthesize new biomass (secondary production) and their activities may be stimulated by fresh OM that induces additional degradation of indigenous OM (i.e., priming effect). Fresh OM can either be delivered or locally produced through cell lysis and exudates from newly produced heterotrophic or chemoautotrophic biomass. It is largely unknown how these processes quantitatively affect the fate and composition of OM (*4*, *8*, *9*). Continuous microbial reworking of OM results in secondary production, but its quantitative contribution to the OM pool is still under debate (*4*, *10*–*13*). For example, Gong and Hollander (*10*) identified substantial microbial contribution to sedimentary OM, while Hartgers *et al.* (*11*) reported only a minimal contribution based on bacterial biomolecular and isotopic signatures.

The priming effect usually refers to enhanced mineralization of OM of the native system (e.g., sediment) due to the input of fresh carbon (*14*). This concept has been intensively explored in terrestrial ecosystems (*14*–*16*) but received little attention in aquatic systems (*17*–*20*), let alone its quantification and the identification of the microbial players involved. This is unexpected because marine sediment continuously receives fresh OM through the deposition of OM produced in the photic zone. Ongoing and projected environmental changes such as the expansion of oxygen minimum zones, sea ice melting, or glacier retreat due to global warming will likely increase the deposition of fresh OM (*21*, *22*) and thus the priming potential. Moreover, benthic animals inhabiting marine surface sediments mix fossil sedimentary OM with recently delivered high-quality OM and thus may stimulate microbial communities (*3*, *23*). Subsurface sediments could potentially receive fresh, labile OM through dead cell lysis (necromass) or cell exudates/lysates of active microbial communities and deep mixing due to bottom trawling or extreme storms. Marine sediments can thus be considered a model system for studying the interactions between OM degradation, priming, and secondary production, but no study to date has quantified these processes simultaneously or for the same location.

Here, we simultaneously studied secondary production, degradation, and priming by adding either fully ^13^C-labeled algal lipid mixture or fully ^13^C-labeled crude proteins to sediment samples from the Helgoland mud area and incubated these samples as well as unamended controls for 400 days. By combining biogeochemical data including dissolved inorganic carbon (DIC), amino acids, microbial lipids, and their isotopic compositions and microbiological information [16*S* ribosomal RNA (rRNA) stable isotope probing (SIP)], we quantified OM degradation, secondary production, and the priming effect in marine sediments and identified the microbial communities involved in each process. Our results show that secondary production and priming are two crucial, overlooked processes that should be incorporated into our conceptual view of sedimentary OM transformation.

## RESULTS AND DISCUSSION

### Algal lipids degrade easily, while algal proteins tend to be preserved

**Fig. 1.**
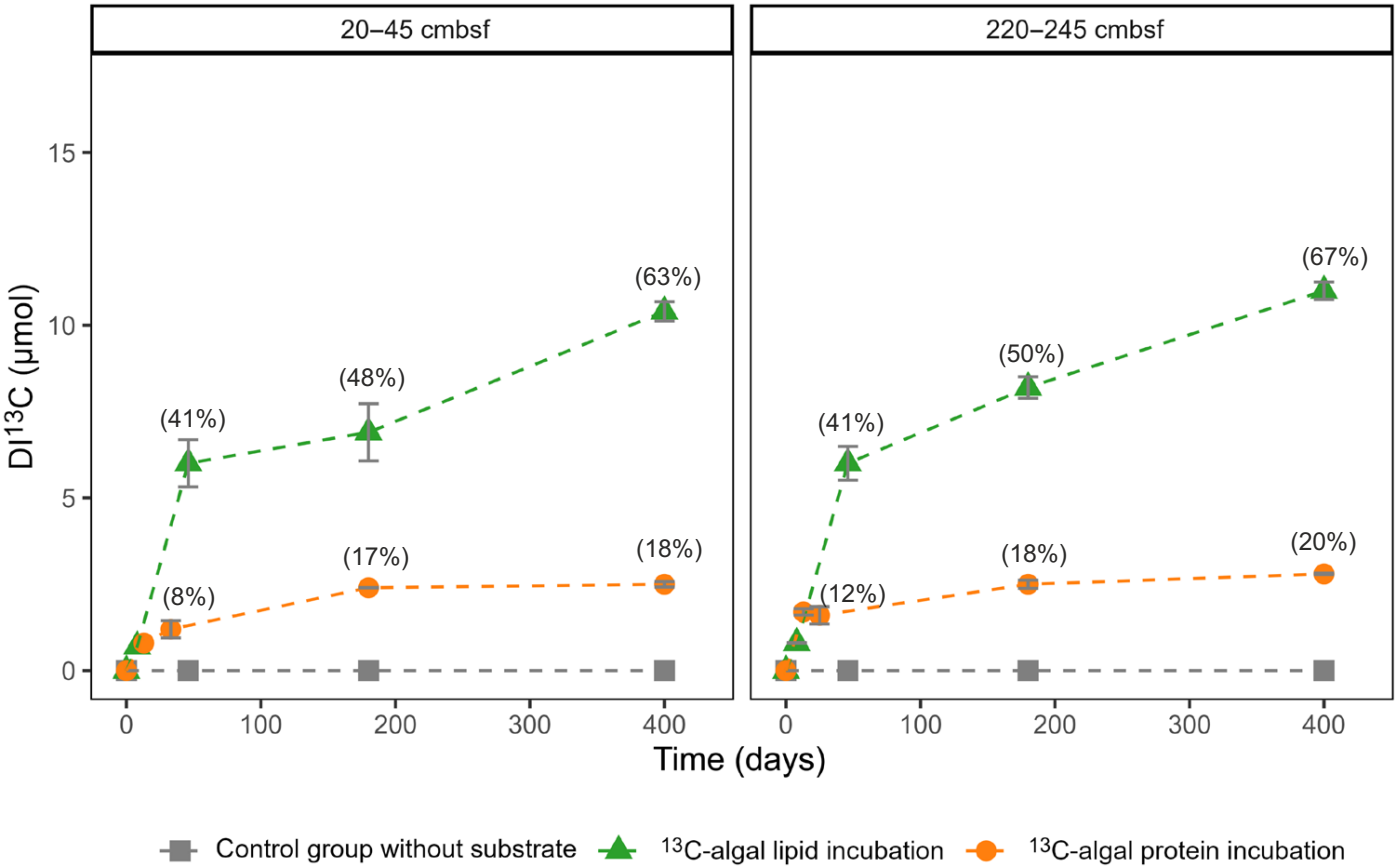
Turnover of organic substrates during 400-day-long incubations. The production of DI^13^C indicates the mineralization of algal substrates (fully ^13^C-labeled algal lipid mixture and fully ^13^C-labeled algal crude proteins) in Helgoland mud area sediment. The percentages in brackets indicate the mineralized substrate fraction.

Sediments from 20 to 45 and 220 to 245 cm below seafloor (cmbsf) were selected for slurry incubation, representing the sulfate-bearing zone and sulfate-depleted zone, respectively. Degradation index (*24*) values are 0.1 and 0 for 20 to 45 and 220 to 245 cmbsf, respectively, indicating similar reactivity. Sediment slurries (dry weight, 5 ± 0.5 g) containing 4700 μmol of organic C (SD, 500 μmol; *N* = 42) were incubated at 10°C under anoxic conditions in triplicate for each harvest time point, either with fully ^13^C-labeled algal lipid mixture (16.5 μmol of ^13^C) or ^13^C-labeled algal crude proteins (14.2 μmol of ^13^C) or without substrate additions (control group). However, 3.7 ± 0.2 μmol of ^13^C of the algal crude protein extract was not hydrolyzable with 6 M hydrochloric acid for 15 hours and appeared as black particles, suggesting that 26% of the proteins were inaccessible. Mineralization, the transformation of organic into inorganic carbon, is widely considered the dominant process in marine sediments (*6*). By measuring the δ^13^C values of headspace CO_2_ and CH_4_, we monitored the mineralization of substrates (fig. S1) and investigated in detail at five different phases including the start of the incubation (day 0 right after the addition of substrates), exponential phase (day 8 or 13), stationary phase (day 33 or 46), mid-term phase (day 180), and long-term phase (day 400). Methanogenic activity was not obvious because CH_4_ was below the detection limit throughout the whole experiment. By measuring the concentration and isotopic composition of DIC (figs. S2 and S3), we quantified the mineralization of ^13^C-labeled organic substrates (DI^13^C; Fig. 1; for calculations, see the Supplementary Materials). In total, 10.4 μmol (20 to 45 cmbsf) and 11 μmol (220 to 245 cmbsf) ^13^C were mineralized from algal lipids after 400-day incubation, which equals 63 and 67% of the introduced ^13^C-algal lipids, respectively (Fig. 1). The general pattern of high-resolution ^13^C-CO_2_ record (fig. S1) reflected two stages of decomposition, i.e., an initial rapid increase during the first 70 days (high reactivity), followed by a slower gradual increase (low reactivity) (*25*). In contrast to lipids, only 2.5 μmol (20 to 45 cmbsf) and 2.8 μmol (220 to 245 cmbsf) ^13^C of algal crude proteins were mineralized, which equals 18 and 20% of the amount of ^13^C introduced, respectively. This limited mineralization of algal protein may be due to its poor quality (nondissolvable black powder with 26% of the carbon being acid nonhydrolyzable) and interactions with the mineral surfaces (*2*, *4*, *24*). Using similar algal crude protein, Pelikan *et al.* (*26*), however, observed substantial protein degradation during incubation of subarctic sediments. These differences, in response, may be due to the one order of magnitude higher substrate addition and much shorter incubation periods in that study. The difference between lipid and protein degradation is consistent with findings from a previous study (*4*). First-order degradation rate constants for the added lipids (0.9 to 1 year^−1^) and proteins (0.18–0.2 year^−1^) are within the range of values reported for marine sediments (*2*, *27*–*29*). Moreover, mineralization rates are similar at the two different depths investigated, despite differences under the biogeochemical conditions (anoxic with or without sulfate) and microbial community composition (see below about RNA-SIP). This supports the rationale underlying the G model of OM degradation according to which the quantity and quality of the substrate are the single most important factors governing OM mineralization rates (*25*, *28*–*30*).

Protein mineralization appeared to be limited after initial degradation because the DI^13^C increase was small. Supporting evidence comes from the ^13^C pattern of the hydrolyzable amino acids: These were rather constant during the whole incubation process (fig. S4). This apparently limited dynamics of amino acids in the protein addition experiment is either due to new biomass synthesis compensating for losses or due to interactions with minerals, lowering the reactivity of amino acids (*31*, *32*). This result is consistent with a soil incubation study, which found that up to 95% of amino acids accumulated as nonmetabolizable soil OM (*33*) and with the long lifetime of bacterially derived amino acids in marine sediments (*4*, *8*, *9*). On extended timescales, organic carbon persisting over millions of years in marine sediments, and presumably stabilized by adsorption to mineral surfaces, has been found to have a proteinaceous nature dominated by amide and carboxyl groups (*34*).

### Biomass derived from secondary production is subject to dynamic transformation

**Fig. 2.**
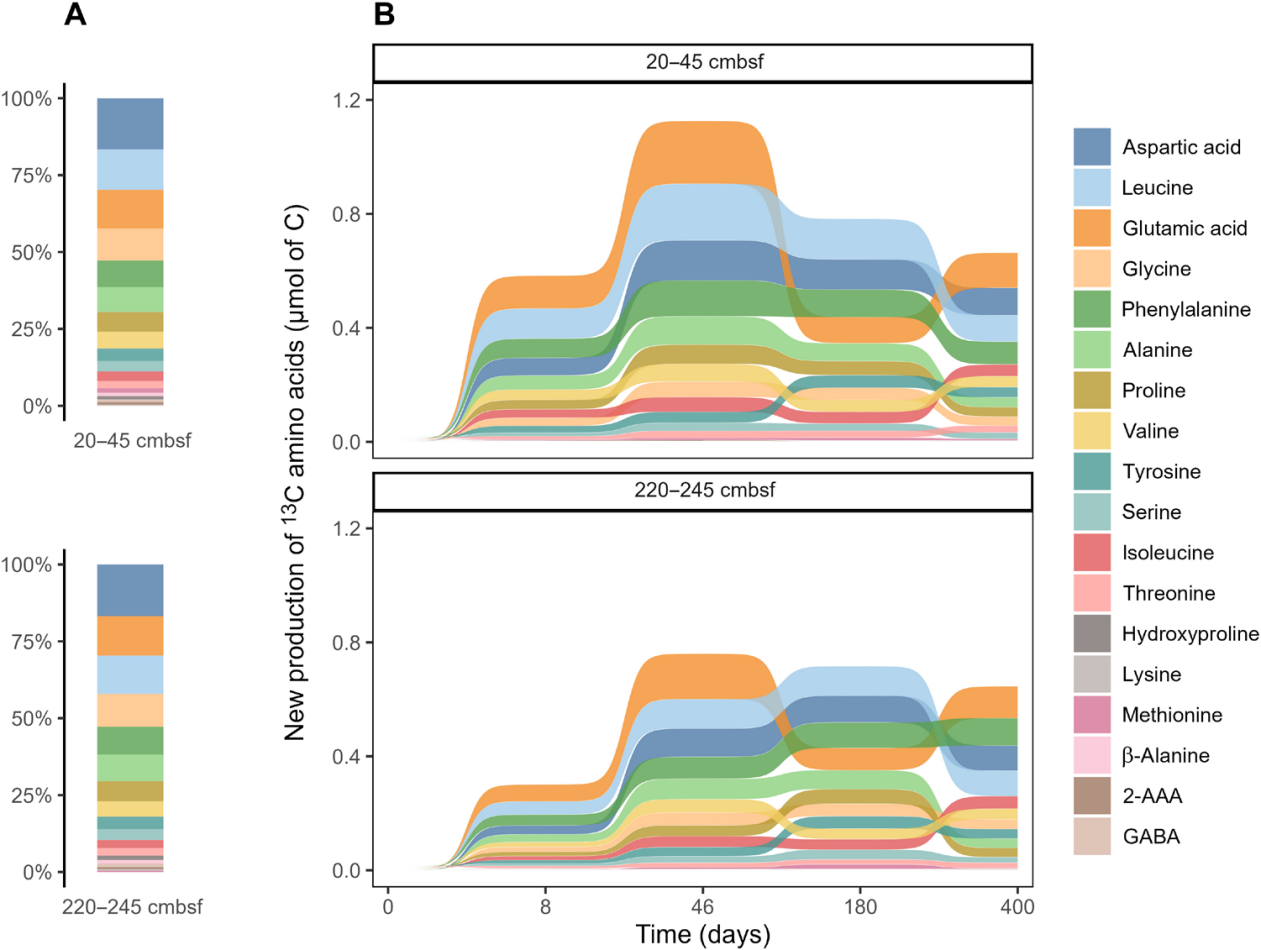
Amino acid distributions indicate microbial secondary production based on added lipid substrate. The relative composition of amino acids in the original Helgoland mud area sediments (**A**) and the new production of amino acids derived from ^13^C-algal lipid mixture at different harvest times (**B**). All the amino acids were normalized to “micromoles of C” to account for different carbon numbers. GABA, γ-aminobutyric acid; 2-AAA, alpha-aminoadipic acid.

**Fig. 3.**
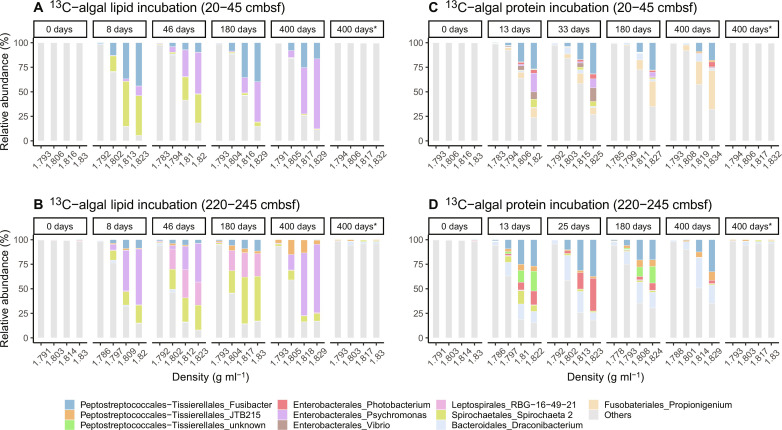
Bacterial communities involved in lipid and protein degradation. (**A** to **D**) RNA-SIP density gradient fractions indicating the active bacteria community involved in the transformation of ^13^C-algal lipid mixture and ^13^C-algal crude proteins at different depths of Helgoland mud area sediments. Asterisk indicates the control experiment without substrate addition.

Secondary production is defined as the generation of biomass by heterotrophic communities and may potentially impact the composition of sedimentary OM (*3*). The natural abundance of hydrolyzable amino acids showed a similar pattern between the two depths in Helgoland sediments (Fig. 2A). We assessed secondary production by quantifying the newly produced ^13^C-labeled amino acids (see the Supplementary Materials). We found that 0.65 μmol ^13^C of algal lipids were transformed into proteins by day 400 (Fig. 2B). If we assume that proteins account for 60% C of microbial biomass (*35*), then this equals 1.08 μmol ^13^C of algal lipids being transformed into the biomass of secondary producers (^13^C_B_ in Table 1). The highest amount of secondary production was observed at the end of the exponential phase (day 46) when 1.85 μmol of ^13^C was transformed into biomass (Table 1). This means that up to 11% of ^13^C-labeled algal lipids was transformed into the biomass of secondary producers. By combining this with mineralization data (Fig. 1), we calculated the carbon use efficiency (CUE; Table 1), i.e., the amount of microbial biomass synthesized per unit of consumed substrate (*36*). On day 8, we observed the highest CUE values of 0.58 and 0.37 for 20 to 45 and 220 to 245 cmbsf, respectively. These values are close to the maximum CUE (~0.60) based on theoretical thermodynamic calculations (*37*) and suggest that half of the carbon consumed from ^13^C-algal lipids was channeled into biomass synthesis at the beginning of incubation. The high CUE values are consistent with RNA information that revealed substantial growth of opportunistic bacteria stimulated by the labile algal lipids (see below about RNA and Fig. 3). Afterward, CUE values decreased to 0.24 (20 to 45 cmbsf) and 0.17 (220 to 245 cmbsf) on day 46 when we observed the largest biomass accumulation indicated by the increase of newly produced amino acids (Fig. 2). CUE values continued to decrease until the end of incubation, reaching 0.09 for both depths. The sustained growth of secondary producers affected not only the total amount of ^13^C in amino acids but also its distribution among individual amino acids (Fig. 2B). This suggests that the newly synthesized biomass was subject to further turnover, consistent with the concept of multiple cycles of OM processing in marine sediments (*3*). This concept is supported by our RNA-SIP analysis that shows a continuously changing composition of active microbial communities (Fig. 3).

**Table 1.** Summary of substrate degradation, priming, secondary production, and CUE in the ^13^C algal lipid incubation. DI^13^C indicates the degradation of ^13^C-algal lipids; DI^12^C indicates the degradation of indigenous OM, including priming and natural degradation (control group). ^13^C_B_ indicates the production of ^13^C-labeled new biomass (in micromoles); ^12^C_B_ indicates the estimated production of new biomass derived from the degradation of indigenous OM (in micromoles); CUE = ^13^C_B_ / (^13^C_B_ + DI^13^C) at time *t*. For detailed calculations, see the Supplementary Materials.

Time (days)	DI^13^C (μmol)	DI^12^C priming (μmol)	DI^12^C control (μmol)	^13^C_B_ (μmol)	^12^C_B_ (μmol)	CUE
*20–45 cmbsf*						
8	0.7	1.3	0.7	0.95	2.71	0.58
46	6	3	4.0	1.85	2.16	0.24
180	6.9	0.6	16.5	1.28	3.17	0.16
400	10.4	30.6	21.0	1.08	5.36	0.09
*220–245 cmbsf*						
8	0.8	8.2	2.1	0.47	6.05	0.37
46	6	29.5	12.0	1.23	8.51	0.17
180	8.2	30.8	22.0	1.17	7.53	0.12
400	11	84	16.0	1.05	9.55	0.09

To identify active microorganisms involved in the processing of isotopically labeled substrates, we separated microbial RNA density into isotopically “light” and “heavy” gradient fractions to select ^13^C-enriched RNA fractions (Fig. 3 and figs. S5 and S6). When microbes prefer ^13^C-labeled substrates over ^12^C background OM, their RNA tends to be more enriched in the heavy-density fraction (*38*). Conversely, when they prefer ^12^C background OM over ^13^C-labeled substrates, their RNA tends to be more enriched in the light or middle-density fractions. *Fusibacter*, Spirochaeota, and *Psychromonas*, which were the numerically minor groups in the original sediments, were immediately stimulated and became the dominant members in algal lipid and protein incubations (highly enriched in the heavy-density fraction; Fig. 3, A to D). These heterotrophs are widely distributed in marine environments (*26*, *39*–*41*) and synthesize specific enzymes targeted to indigenous OM. On the basis of metagenome-assembled genomes from previous studies, these microbes have the potential to catabolize either lipids or proteins via extracellular hydrolases (peptidases, lipases, etc.) (*26*, *41*). Leptospirales RBG-16-49-2 became prominent only in the deeper sediments when ^13^C-algal lipids were added. Peptostreptococcales-Tissiereilales JTB215 initially had a minor contribution but gradually increased to up to 9% in the heavy fraction at the end of the incubations. ^13^C-algal proteins stimulated additional bacteria including members of the genera *Propionigenium*, *Draconibacterium*, and *Photobacterium* (Fig. 3, C and D). Given their relatively high abundance, these three groups play an important role in protein degradation. The abundance of most of these heterotrophs was below 1% in the control without substrate addition. It confirms that fresh OM input substantially affects the numerically minor microbial groups that may play a more important role in OM transformation than the more abundant members in marine sediments.

The role of archaea remains an interesting question concerning their possible contribution to OM transformation in marine sediments (*42*, *43*). Archaea seem to play a less important role than bacteria in processing algal lipids and protein based on the low ^13^C incorporation into archaeal lipids and RNA (figs. S7 and S8). However, considering the slow turnover of archaeal lipids and the influence of fossil archaeal lipid pools (*44*, *45*), their specific role in processing OM in marine sediments needs more evaluation. We observed very different archaeal communities between 20 to 45 and 220 to 245 cmbsf, showing their adaptation to different biogeochemical conditions (fig. S8). Asgardarchaeota, Woesearchaeales, and SG8-5 showed active signals based on their slight enrichment in heavy-density RNA fractions (fig. S8). Their low abundance indicates that the numerically minor members of the archaeal community participate in OM turnover. In contrast, the majority of archaea displayed similar RNA abundance between the light and heavy fractions, including Bathyarchaeota and Lokiarchaeota. Although they are not involved in the direct degradation of algal lipids and proteins, their relative abundance varying with time suggests that they might use sedimentary OM or their fermentation products. Previous studies have also shown that Bathyarchaeota and Lokiarchaeota can metabolize various organic molecules based on their physiology and genomes (*43*, *46*, *47*). We did not observe the growth of methanogens (fig. S8) or the production of methane in the sediment incubations, indicating that algal lipids and proteins as well as their fermentation products were not used for methanogenesis. Subsurface sediments of the Helgoland mud area are rich in reactive iron oxides and have high dissolved iron concentrations, suggesting that iron-reducing microbes are involved in mineralization (*48*).

### Substantial priming is mediated by diverse heterotrophic bacteria and sulfur- and iron-cycling bacteria

**Fig. 4.**
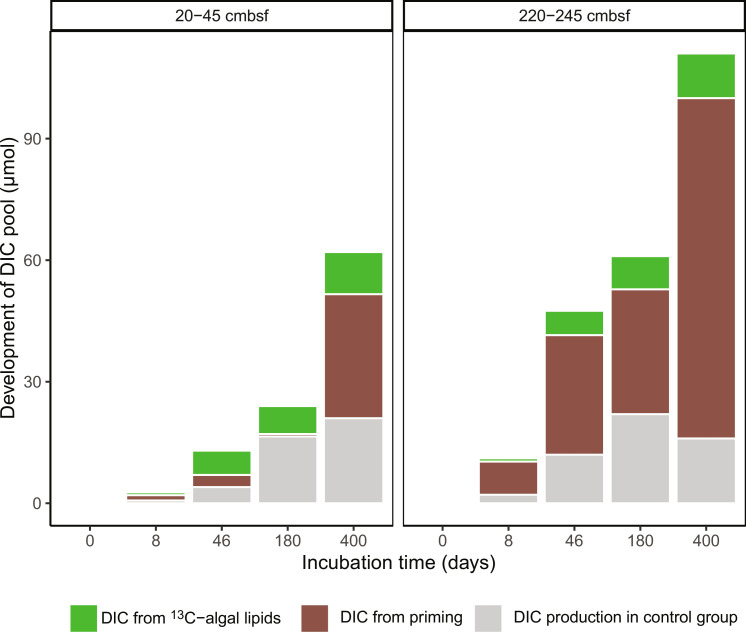
Differentiating the sources of evolved DIC during incubations with ^13^C-algal lipid mixture as substrate. DIC from ^13^C-algal lipids indicates the mineralization of substrate. DIC from priming indicates the accelerated mineralization of indigenous OM. DIC production in the control group indicates the natural mineralization of indigenous OM.

By measuring the DIC concentration and stable isotope changes (figs. S2 and S3), we differentiated between the production of DI^12^C and DI^13^C (see the Supplementary Materials), with the former resulting from the mineralization of indigenous OM. This allowed us to quantify the priming effect as the difference in DI^12^C production in treatments with and without substrate addition (*14*, *15*). Priming was substantial in the ^13^C-algal lipid incubations where DI^12^C increased faster than that in the control (Fig. 4, Table 1, and fig. S9), particularly in the deeper sediment and after the stationary phase. Compared to the control group, extra DI^12^C was observed in the ^13^C-algal lipid incubation already on day 8, indicating the triggering of priming after the supply of fresh OM. Throughout the experiment, this additional production of DI^12^C continued, indicating a long-term effect of the added substrate. After 400 days, the addition of algal lipids enhanced the mineralization of background OM by a factor of 2.5 (20 to 45 cmbsf) and 6 (220 to 245 cmbsf). This is substantially larger than the previous quantification in coastal sediments (31%) (*19*) and the average of 802 soils globally (42%) (*15*) but similar to some aquatic environments (up to 500%) (*49*) and soils (up to 400%) (*50*). Such high priming effect and its longevity over more than 1 year imply that it plays an important role in carbon cycling in marine sediments that frequently receive fresh OM (primary input to surface sediments and secondary input to subsurface sediments) or that are regularly subject to physical or biological particle mixing (*3*, *19*, *23*). We could not differentiate total DIC production between ^13^C-algal protein incubation and the control group (fig. S3), indicating that algal protein stimulated no obvious priming. This may be partly caused by the quality of the added algal protein that is a nondissolvable black powder with 26% of the carbon being acid nonhydrolyzable. Moreover, the potential mineral-organic associations may also inhibit their priming effects.

Although the heterotrophic bacteria dominating the ^13^C-enriched fractions preferentially use fresh ^13^C-labeled OM (lipids and proteins), their ability to use indigenous OM cannot be excluded because of their enrichment in the middle-density fraction of RNA when compared to the control (Fig. 3). The respective microbes likely received energy from fresh OM degradation to secret extracellular enzymes for the degradation of indigenous sedimentary OM. This priming effect is commonly observed in soils (*14*, *51*) but is often neglected in marine sediments.

**Fig. 5.**
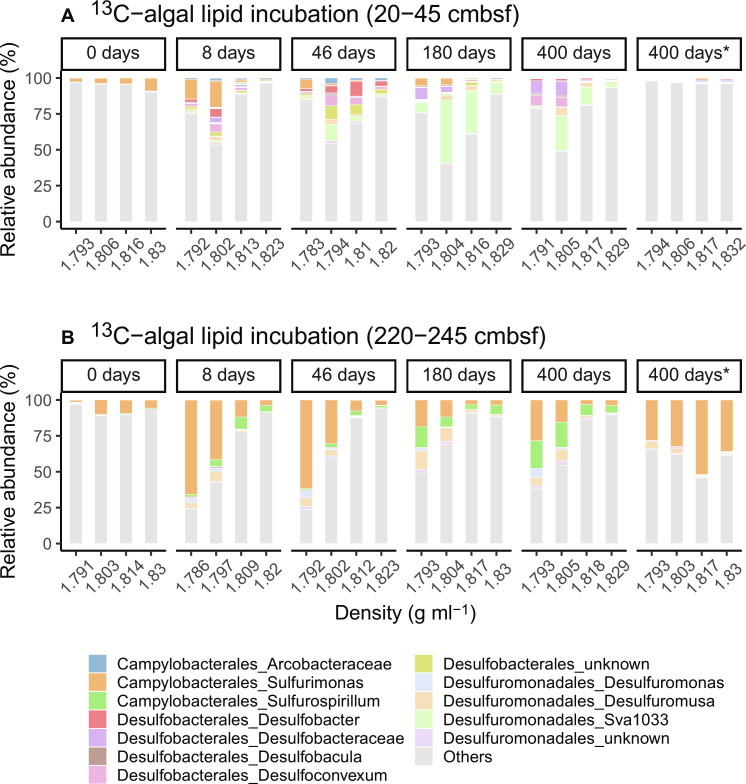
Bacterial communities involved in priming. RNA-SIP density gradient fractions indicating active bacteria community involved in priming in two sediment depths [20 to 45 cmbsf in (**A**) and 220 to 245 cmbsf in (**B**)]. Asterisk indicates the control experiment without substrate addition.

When microbes prefer natural, ^12^C background OM over added ^13^C substrates, their RNA tends to be more enriched in the light or middle-density fractions (*38*). Moreover, an increasing abundance in the light and middle fractions would be expected with incubation time (compared to day 0) because of continuous microbial processing of ^12^C. On the basis of these two principles, we inferred the identities of microbial players involved in the priming effect as specific as down to the genus level (Fig. 5). In the incubation with ^13^C-algal lipids, we detected diverse bacterial groups that were highly enriched in the light and middle fractions such as Campylobacterales, Desulfobacterales, and Desulfuromonadales. In these orders, the most dominant bacteria were Sva1033 (family level) and *Sulfurimonas* (genus level). Sva1033 tends to be active in dissimilatory iron reduction in marine sediments (*52*). Other iron-reducing bacteria such as Desulfuromonas and Desulfuromusa were also identified in our incubations, and they use fermentation products such as acetate, lactate, propionate, formate, or hydrogen as electron donors (*53*). The activity of these iron-related microbes was supported by the high reactive iron oxide content of these sediments (*48*). *Sulfurimonas* can degrade hydrocarbons although they are mostly considered sulfur-oxidizing autotrophs (*54*, *55*). Sulfate and sulfur reducers such as Desulfobacterales and Desulfuromonadales might use fermentation products (e.g., volatile fatty acids) derived from both algal substrates and sedimentary OM (*26*). Except for *Sulfurimonas*, all microbes putatively involved in priming account for less than 1% in the unamended controls. They preferentially use sedimentary OM by cometabolism with the fresh OM degraders identified in this study (Figs. 3 and 5). This suggests that the shifts and succession of the degrader community structure (both fresh and refractory OM degraders) and their collective activities are important drivers of the priming effect. Nonetheless, caution should be taken because slurry experiments change the original structure of sediments by dilution and mixing, the interaction between substrate and mineral surfaces, and may affect the interrelationships among microbes that jointly degrade complex OM.

### Degradation, secondary production, and priming interact with each other

We explored two understudied processes of OM transformation, secondary production, and priming. When fresh OM (phytodetritus, necromass after cell lysis, etc.) is introduced into the sedimentary system, microbes respond immediately by changing their abundance and diversity (Fig. 3 and fig. S6). These degraders secrete extracellular enzymes to catalyze the breakdown and mineralization of not only fresh OM but also sedimentary OM (Table 1 and Figs. 1 and 4). Meanwhile, they synthesize new biomass, which contributes to secondary production. We quantified the secondary production from the ^13^C-algal lipids (Fig. 2) but did not take into account the contribution from sedimentary OM. The mineralization of indigenous sedimentary OM is 5 to 10 times higher than that of added ^13^C-algal lipids (Fig. 4). Assuming the same CUE for indigenous and added substrates, we would expect 5 to 10 times higher secondary production based on indigenous sedimentary OM (^12^C_B_ in Table 1 and the Supplementary Materials). Consequently, the impact of secondary microbial production on OM composition is larger than can be inferred from ^13^C incorporation in new microbial biomass. However, the new biomass formed through secondary production is subject to further transformation, possibly stimulating more priming over a longer period. This can be identified through the shifts and succession of microbial community structures (Figs. 3 and 5), amino acid distribution (Fig. 2), and enhanced indigenous OM mineralization (Fig. 4). Multiple cycles of OM processing (degradation, secondary production, and priming) by bacteria eventually contribute to the formation of molecularly uncharacterizable OM, which accounts for up to 80% of the sedimentary OM (*3*, *5*, *56*). The two understudied processes, secondary production and priming, should be incorporated into our understanding of OM cycling in marine sediments. Sediments without biological mixing of indigenous and fresh OM are known to less efficiently transform and mineralize OM and hence bury more OM (*3*, *23*, *30*). Global warming–induced environmental changes such as sea ice melting increase the delivery of fresh OM to the seafloor (*57*) and thus raise the potential for secondary production and priming. Our study revealed that the addition of labile substrates induces not only additional mineralization but also additional secondary production based on indigenous OM. This additional secondary production has hitherto not been taken into consideration in our conceptual and mathematical models, and, thus, its importance in other ecosystems and at the global scale requires further study.

## MATERIALS AND METHODS

### Sediment incubation

The sediment material for the slurry incubations was obtained from gravity cores recovered in the Helgoland mud area (North Sea; 54°05.23′N, 007°58.04′E; RV HEINCKE HE483; water depth, 28 m). The cores were separated into 25-cm sections on board and stored anaerobically at 4°C in 2.6-liter jars with anoxic artificial seawater [NaCl (26.4 g liter^−1^), MgCl_2_·6H_2_O (11.2 g liter^−1^), CaCl_2_·2H_2_O (1.5 g liter^−1^), and KCl (0.7 g liter^−1^)]. Sediments from sections 20 to 45 and 220 to 245 cmbsf were selected and mixed with anoxic artificial seawater (w/v, 1:4) in sterile 120-ml serum bottles that were sealed with butyl rubber stoppers. The headspace was flushed with N_2_ to assure the anoxic condition. Sediment at 20 to 45 cmbsf represented the sulfate bearing zone, and its slurries were supplemented with sulfate to a final concentration of 10 mM to mimic in situ conditions. Sediment at 220 to 245 cmbsf was sulfate-depleted and supported iron reduction (*48*).

Incubation series were prepared with sediment slurries (dry weight, 5.0 ± 0.5 g), containing 4700 μmol of organic C (SD, 500 μmol; *N* = 42), in triplicate per each harvest time point. Series “^13^C-algal lipid incubation” was supplemented with 500 μg of ^13^C-labeled algal lipid mixture [99 atom % of ^13^C, ISOTEC, Sigma-Aldrich, oily and dark green solid that can be fully dissolved in the dichloromethane (DCM)] with a carbon content of 43.1 ± 0.22% (*n* = 6) that equals 16.5 μmol of ^13^C. Series “^13^C-algal protein incubation” was supplemented with 500 μg of ^13^C-labeled algal crude proteins (99 atom % of ^13^C, ISOTEC, Sigma-Aldrich, black powder and nondissolvable in water) with a carbon content of 36.9 ± 0.20% (*n* = 6) that equals 14.2 μmol of ^13^C. Both algal lipid mixture and crude proteins are extracted from cyanobacteria. However, after treatment of the algal crude proteins with 6 M hydrochloric acid for 15 hours, we observed that 3.7 ± 0.2 μmol of ^13^C still stayed nonhydrolyzable as black particles. This means that 26% of the carbon from algal crude protein is inaccessible. Sediment slurries without substrate addition were also incubated as a control group. The incubations were carried out at 10°C temperature under anoxic conditions. Incubation time was controlled on the basis of the development of δ^13^C values of CO_2_ and CH_4_ in the headspace. δ^13^C of CO_2_ and CH_4_ were measured by Thermo Finnigan Trace GC coupled to a Thermo Finnigan Delta plus XP isotope ratio mass spectrometer (IRMS) and reported in the delta notation as δ^13^C relative to the Vienna Pee Dee belemnite (VPDB) standard. Deviations of triplicate isotopic measurement of δ^13^C of CO_2_ were between ±1 and ±1000 per mil (‰) (with label uptake of >10,000‰). Slurries were harvested at five time points for detailed analyses.

### DIC measurement

Following (*58*), an aliquot of the slurry was filtered with a 0.2-μm filter to remove cells and other particles. After filtration, 1 ml of samples was acidified with 100 μl of 45% phosphoric acid overnight in an Exetainer vial prepurged with CO_2_-free air before analysis. A Delta Ray isotope ratio infrared spectrometer with URI (universal reference interface) Connect (Thermo Fisher Scientific, Germany) was used for analysis of CO_2_ composition in the vial’s headspace that was transferred in total into the system by a Cetac ASX-7100 autosampler. All isotopic values were reported in the delta notation as δ^13^C relative to the VPDB standard with an analytical error of ±1‰ for DIC with natural ^13^C abundance. Deviations of triplicate isotopic measurement of sample DIC were between ±1 and ±1000‰ (with label uptake of >10,000‰). DIC concentration was calculated from the detected total volume of CO_2_ in the headspace by a calibration series prepared with sodium hydrogen carbonate solution.

### Amino acids derivatization and analysis

Sediment slurries were centrifuged to separate the sediments from the medium, freeze-dried, and homogenized. Fifty milligrams of samples, spiked with the internal reference standard norleucine, were decalcified and acid hydrolyzed (6 M at 110°C) for 15 hours. The hydrolysate was centrifuged to remove the sediments and subsequently processed three times with hexane/DCM mixture (2/1, v/v) to remove the lipids. The purification and derivatization of amino acids were performed by following the protocols described by Takano *et al.* (*59*) and Chikaraishi *et al.* (*60*), respectively. Briefly, the hydrolysates were evaporated under N_2_ gas to dryness and purified using Dowex 50WX8 200- to 400-mesh cation-exchange resin to eliminate the matrix effects. The purified amino acids were isopropylated with a mixture of isopropanol and acetyl chloride (4/1, v/v) at 100°C for 2 hours. The solution was evaporated to dryness, followed by three times DCM addition and evaporation to remove any remaining reagent. The amino acid isopropyl esters were then acylated using a mixture of pivaloyl chloride and DCM (1/1, v/v) at 100°C for 2 hours to obtain pivaloyl-isopropyl ester (Pv/AA/iPr). The Pv/AA/iPr solution was evaporated to dryness, followed by three times DCM addition and evaporation to remove any remaining reagent. Liquid-liquid extraction was performed by MiliQ water and a hexane/DCM mixture (2/1, v/v). The Pv/AA/iPr were stored frozen (−20°C) and dissolved in ethyl acetate before analysis. Molar and stable carbon isotopic compositions of amino acids were determined by gas chromatography (GC)–flame ionization detector (Trace GC 2000, Thermo Finnigan, Bremen, Germany) and GC-IRMS (Trace GC Ultra coupled to a Delta V Plus IRMS via GC IsoLink connected to a ConFlow IV interface, Thermo Fisher Scientific, Bremen, Germany). A Restek Rxi-5ms column (30 m × 250 μm × 0.25 μm; Restek, Bad Homburg, Germany) was used for the chromatographic separation of amino acids. The initial GC oven temperature was held at 60°C for 3 min, increased to 110°C at a rate of 15°C min^−1^, increased to 150°C at a rate of 3°C min^−1^, then raised to 220°C at a rate of 6°C min^−1^, and held at 220°C for 17 min. The carrier gas was helium with a constant flow rate of 1.0 ml min^−1^. The injector temperature was set at 290°C. The oxidation oven of the combustion interface was operated at 940°C. All isotopic values are reported in the delta notation as δ^13^C relative to the VPDB standard. Deviations of triplicate isotopic measurement of amino acids were between ±1 and ±5‰ (with label uptake of >100‰). The degradation index was calculated according to Dauwe *et al.* (*24*), and CUE (*36*, *37*) was calculated on the basis of the production of DI^13^C and ^13^C_B_, which was converted from ^13^C amino acids by division through 0.6 (*35*). For detailed calculations, see the Supplementary Materials.

### Isopycnic centrifugation and gradient fractionation of RNA-SIP

RNA was extracted from incubation slurries as described previously (*46*, *61*). Isopycnic centrifugation and gradient fractionation were conducted to separate ^13^C-labeled from unlabeled RNA. In detail, 500 to 1000 ng of RNA was loaded with formamide (240 μl), cesium trifluoroacetate solution (6 ml), and gradient buffer solution, followed by ultracentrifugation using an Optima L-90 XP ultracentrifuge (Beckman Coulter, Brea, CA, USA). After centrifugation at 124,000*g* at 20°C for 65 hours, 14 fractions (~410 μl for each fraction) were collected from each sample. At the same time, mixed fully ^13^C-labeled and unlabeled RNA from *Escherichia coli* was used as standard during density separation for defining heavy and light fractions. cDNA was obtained by reverse transcription of RNA using GoScript reverse transcription kit (Promega, Madison, Wisconsin, USA). A combination of cDNA from fractions 4 and 5 (heavy), 6 and 7 (middle), 8 and 9 (light), and 10 and 11 (ultralight) was performed for 16*S* rRNA gene sequencing, respectively.

### 16*S* rRNA cDNA sequencing

Polymerase chain reaction (PCR) was performed with a barcoded primer pair [bacteria: Bac515F/805R, 5′-GTGYCAGCMGCCG-CGGTAA-3′/5′-GACTACHVGGGTATCTAATCC-3′; archaea: Arc519F/806R, 5′-CAGCMGCCGCGGTAA-3′/5′-GGACTACVS-GGGTATCTAAT-3′) (*62*) using KAPA HiFi HotStart PCR kit (KAPA Biosystems, Cape Town, South Africa). Thermocycling was set as follows: 95°C for 3 min; 35 cycles at 98°C for 20 s, 61°C for 15 s, and 72°C for 15 s; and 72°C for 1 min. Amplicons were sequenced on the NovaSeq 6000 platform [2× 250 base pairs (bp)] at Novogene (Cambridge, UK). The raw reads were analyzed according to Hassenrück (*63*). Briefly, barcodes were extracted, followed by demultiplexing and primer clipping using cutadapt (version 2.1). The demultiplexed reads were then analyzed using dada2 (version 1.16.0). In detail, the quality of sequencing reads was checked, and then the reads were trimmed, followed by the correction of error estimates and error learning to retrieve the final clean reads. The clean reads were then dereplicated and denoised, which were further merged for both forward and reverse reads to obtain the long sequences. The chimera reads were then filtered, and the unusual reads below 248 bp or above 256 bp were removed. Taxonomy was assigned using the final reads based on the database SILVA 138 (*64*).
