# Supplementary material for: Secondary production and priming reshape the organic matter composition in marine sediments
Source: Sci Adv. 2024 May 17;10(20):eadm8096. doi: 10.1126/sciadv.adm8096 (PMC11100564; doi:10.1126/sciadv.adm8096)
Supplement: Supplementary file 1 — Supplementary Text Figs. S1 to S9 [file sciadv.adm8096_sm.pdf]

Science Advances
AAAS

Supplementary Materials for

Secondary production and priming reshape the organic matter composition in marine sediments

Qing-Zeng Zhu *et al.*

Corresponding author: Qing-Zeng Zhu, qzzhu@marum.de

Sci. Adv. **10**, eadm8096 (2024)
DOI: 10.1126/sciadv.adm8096

This PDF file includes:

Supplementary Text
Figs. S1 to S9

Supplementary Text

Quantification of the carbon content of ^{13}C-algal lipids and ^{13}C-algal proteins

To quantify the carbon content of fully ^{13}C-labeled substrates, 2 parts of the substrate were mixed with 98 parts of glucose followed by measurement via EA-IRMS. The carbon content of the substrates was calculated based on equation (S1) and (S2)

$$\delta^{13}C_{mix} \times M_{mix} = \delta^{13}C_{glu} \times M_{glu} + \delta^{13}C_{sub} \times M_{sub} \quad \text{(S1)}$$

$$M_{mix} = M_{glu} + M_{sub} \quad \text{(S2)}$$

with M defined as the weight (mg) of different organic compounds.

Quantification of DIC production at time t

$$\Delta DIC_t = DIC_t - DIC_{t0} \quad \text{(S3)}$$

Quantification of $DI^{13}C$ production at time t

$$DI^{13}C_t = DIC_t \times (f_t - f_0) \quad \text{(S4)}$$

f is defined as the ^{13}C content of DIC:

$$f = \frac{^{13}\text{C}-\text{DIC}}{^{12}\text{C}-\text{DIC}+\ ^{13}\text{C}-\text{DIC}} = \frac{R}{1+\text{R}} \quad \text{(S5)}$$

Quantification of $DI^{12}C$ production at time t

$$DI^{12}C_t = \Delta DIC_t - DI^{13}C_t \quad \text{(S6)}$$

$DI^{12}C_t$ can be calculated from the incubation group and it is the sum of ^{12}C-DIC priming and ^{12}C-DIC control. ^{12}C-DIC control is the DIC production from the control group where no substrate was provided.

Quantification of ^{13}C-amino acids (AA) production at time t

$$AA^{13}C_t = AA_t \times (f_t - f_0) \quad \text{(S7)}$$

f is defined as the ^{13}C content of all amino acids:

$$f = \frac{^{13}\mathrm{C{-}AAs}}{^{12}\mathrm{C{-}AAs} + {}^{13}\mathrm{C{-}AAs}} = \frac{R}{1 + \mathrm{R}} \quad \text{(S8)}$$

Quantification of biomass based on ^{13}C-amino acids (AA) production at time t

$AA^{13}C_t$ equals the newly produced protein and accounts for around 60% of newly produced biomass, ^{13}C-B *(see reference 35)*

$$^{13}C_B = AA^{13}C_t \div 0.6 \quad \text{(S9)}$$

$$^{12}C_B = {}^{13}C_B \times DI^{12}C \div DI^{13}C \quad \text{(S10)}$$

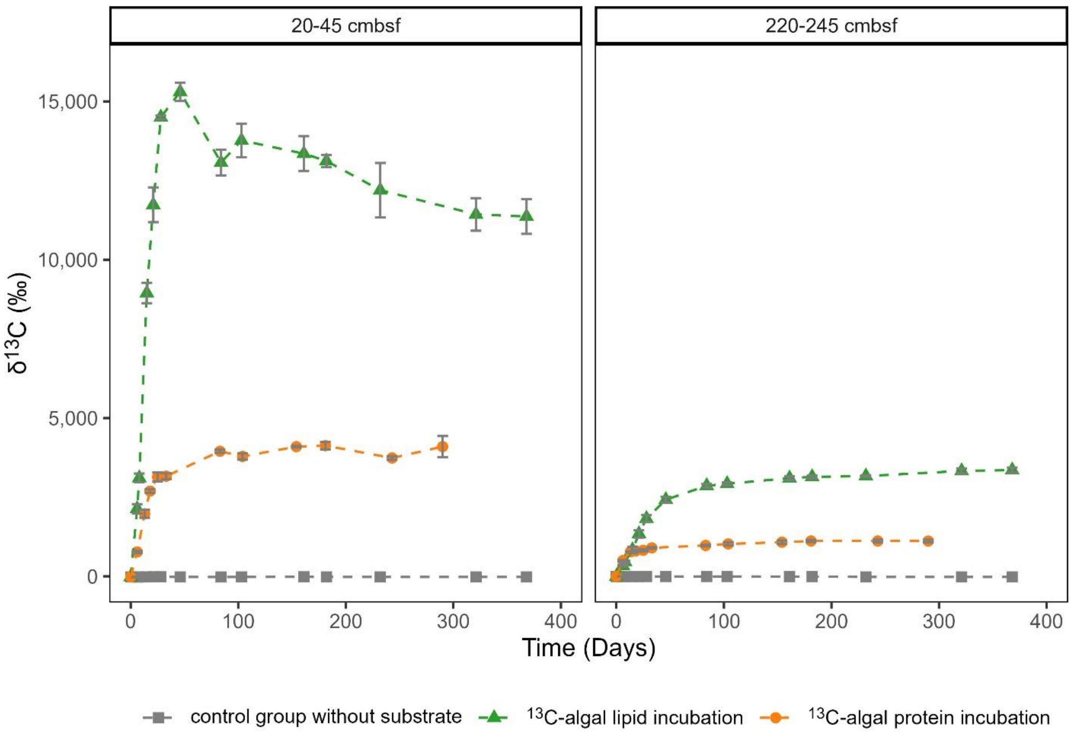

Fig. S1. δ^{13}C of CO_2 indicates the turnover of added organic substrates during 400-day-long incubations. Development of δ^{13}C-CO_2 in the headspace during incubations with algal substrates (^{13}C-algal lipids or ^{13}C-algal proteins) and the control group without substrate in two depths of Helgoland mud area sediment.

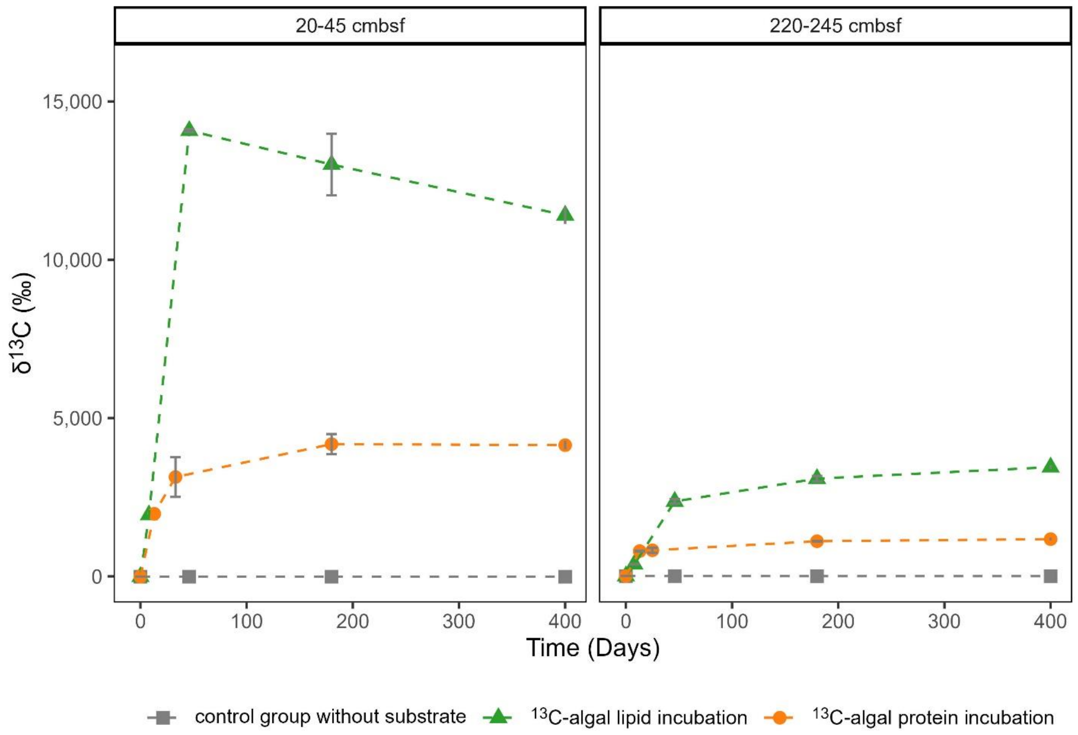

Fig. S2. δ^{13}C of DIC indicates the turnover of added organic substrates during 400-day-long incubations. Development of δ^{13}C-DIC in the liquid phase during incubations with algal substrates (^{13}C-algal lipids or ^{13}C-algal proteins) and the control group without substrate in two depths of Helgoland mud area sediment.

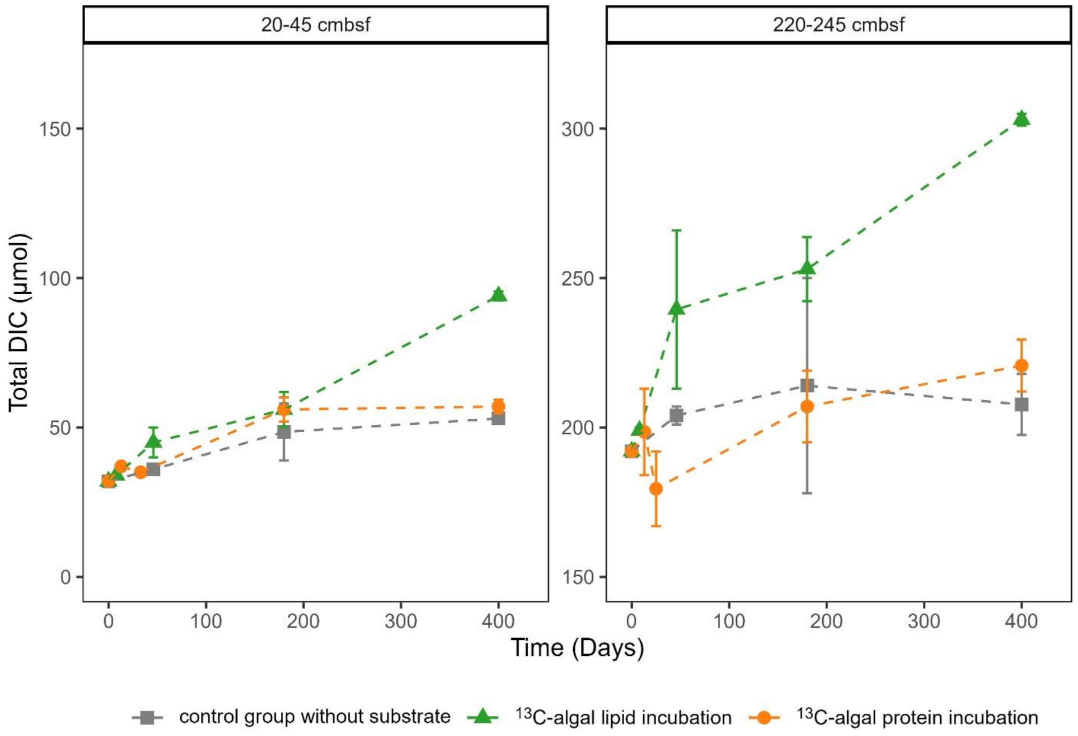

Fig. S3. DIC indicates the turnover of organic matter in the system during 400-day-long incubations. Development of total DIC concentration during incubations with algal substrates (^{13}C-algal lipids or ^{13}C-algal proteins) and the control group without substrate in two depths of Helgoland mud area sediment.

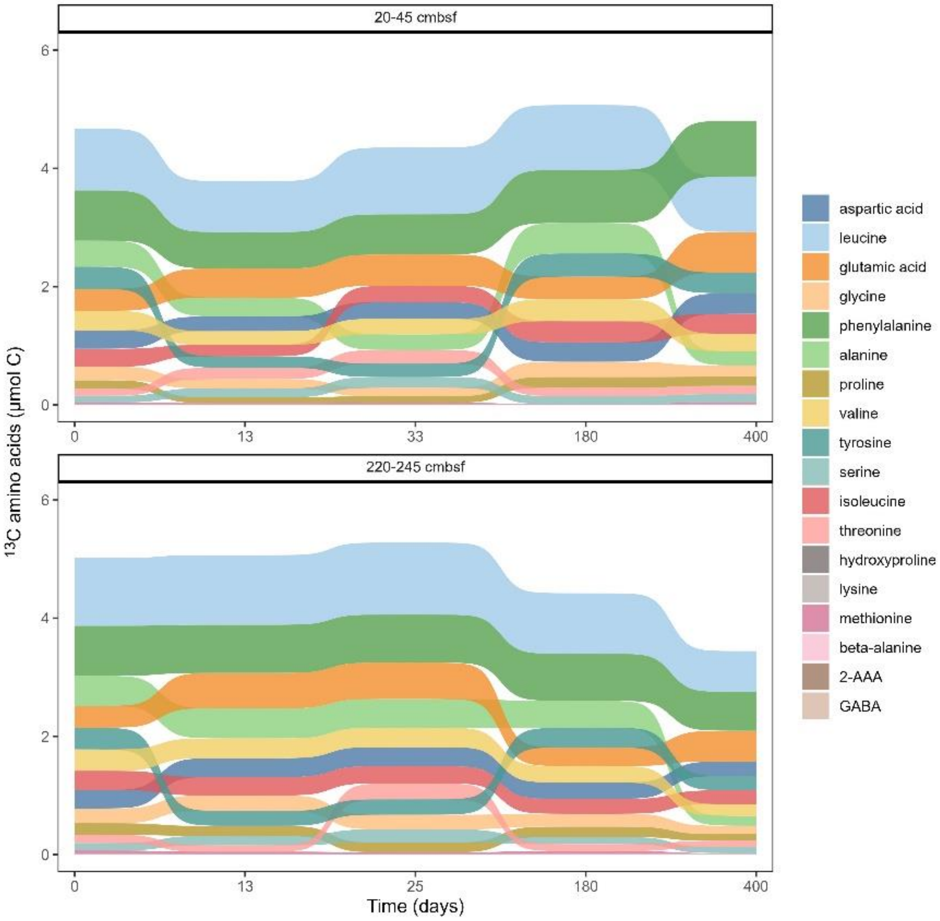

Fig. S4. Distributions of ^{13}C-labeled amino acids indicate mixed signals of substrate turnover and secondary production. The compositional change of ^{13}C-labeled amino acids (normalized to "µmol C" to account for different carbon numbers) during the ^{13}C-algal protein incubations.

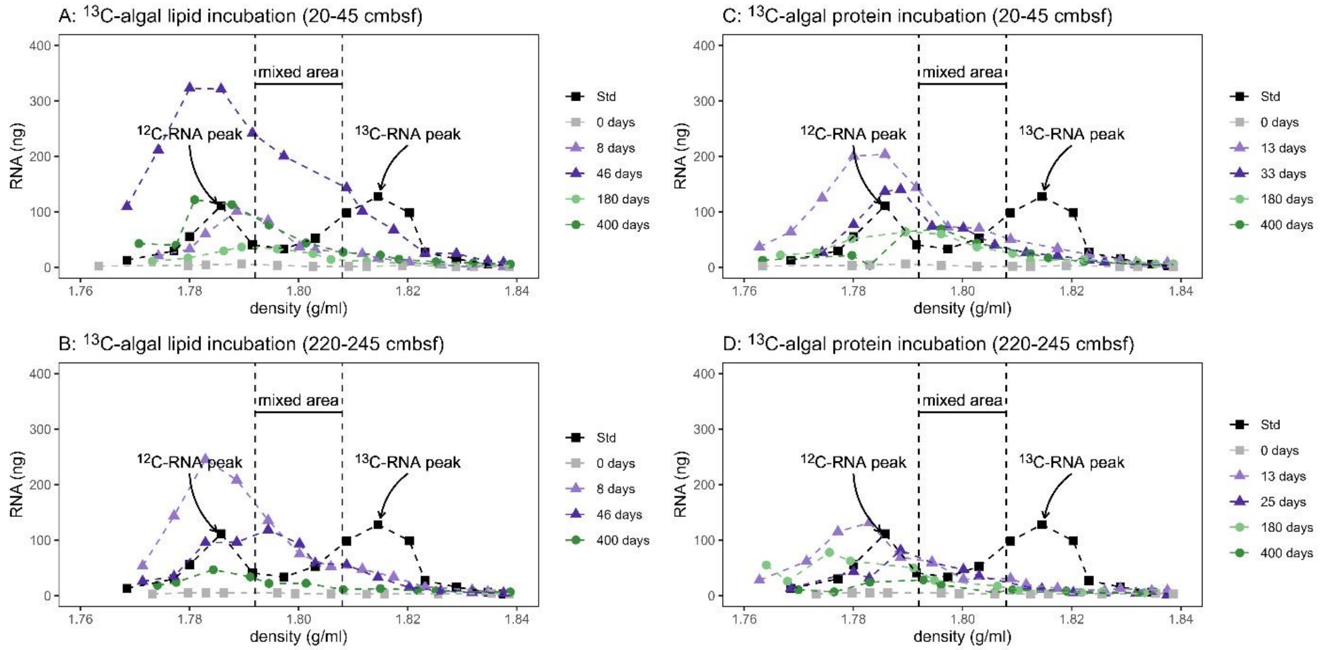

Fig. S5. RNA distribution in different density fractions during the ^{13}C-algal lipid and protein incubations. Std indicates the RNA standard containing an equal amount of ^{12}C- and ^{13}C-labeled nucleic acids from E. coli. The mixed area between vertical dashed lines indicates fractions that are partially ^{13}C-labeled.

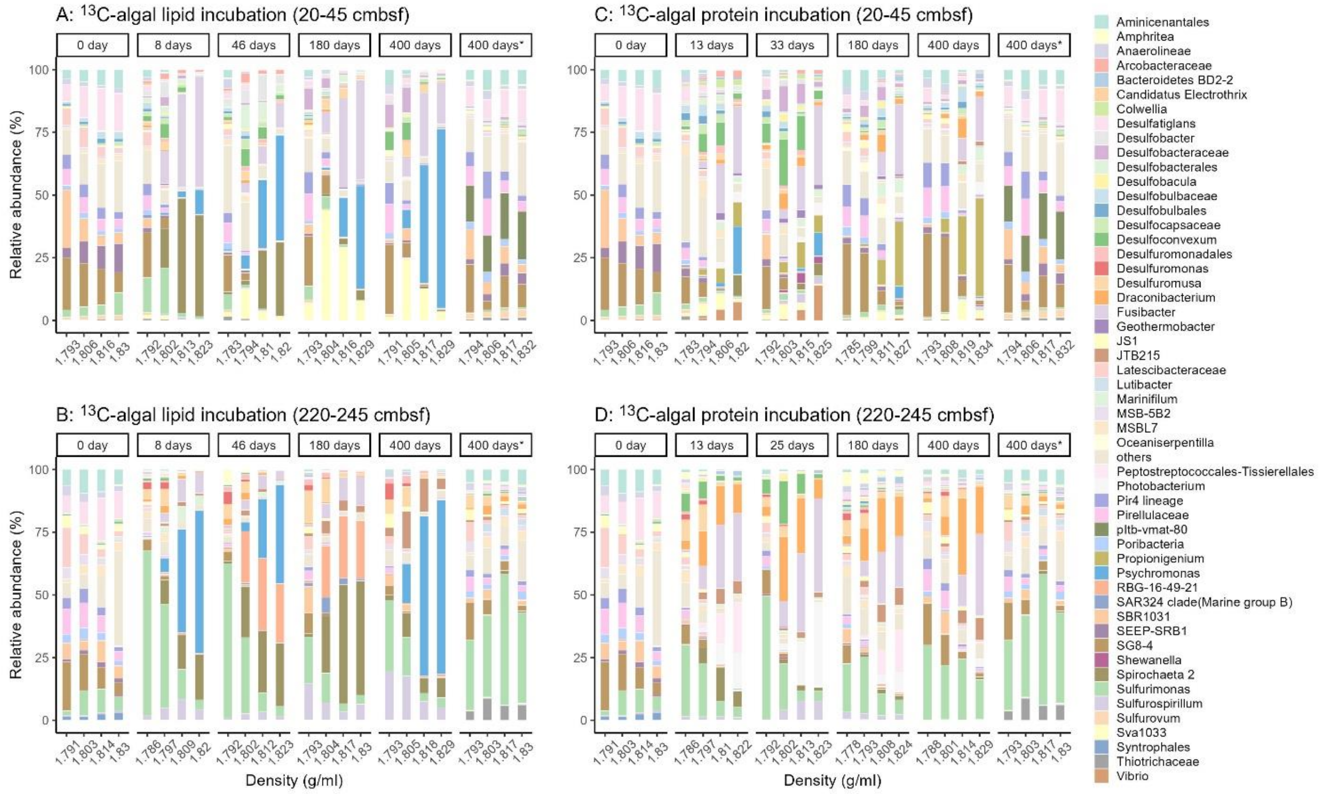

Fig. S6. Bacterial community composition during lipid and protein degradation. RNA density distribution targeting algal lipid and protein bacterial degraders during the ^{13}C-algal lipid and protein incubations.

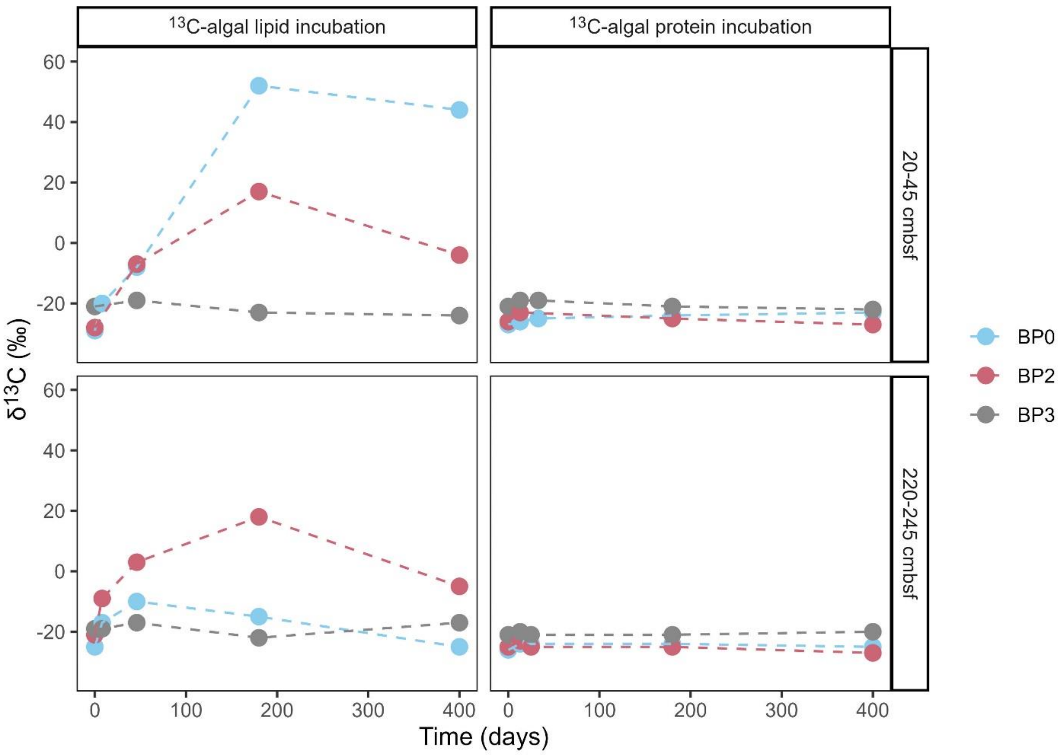

Fig. S7. The δ^{13}C value archaeal lipid derivatives indicate the activity of archaea. The change in carbon isotopic compositions of different biphytanes (BP) derived from archaeal lipids during the ^{13}C-algal lipid and protein incubations in two depths of Helgoland mud area sediment. The number behind BP indicates the number of cyclopentane rings in the molecules.

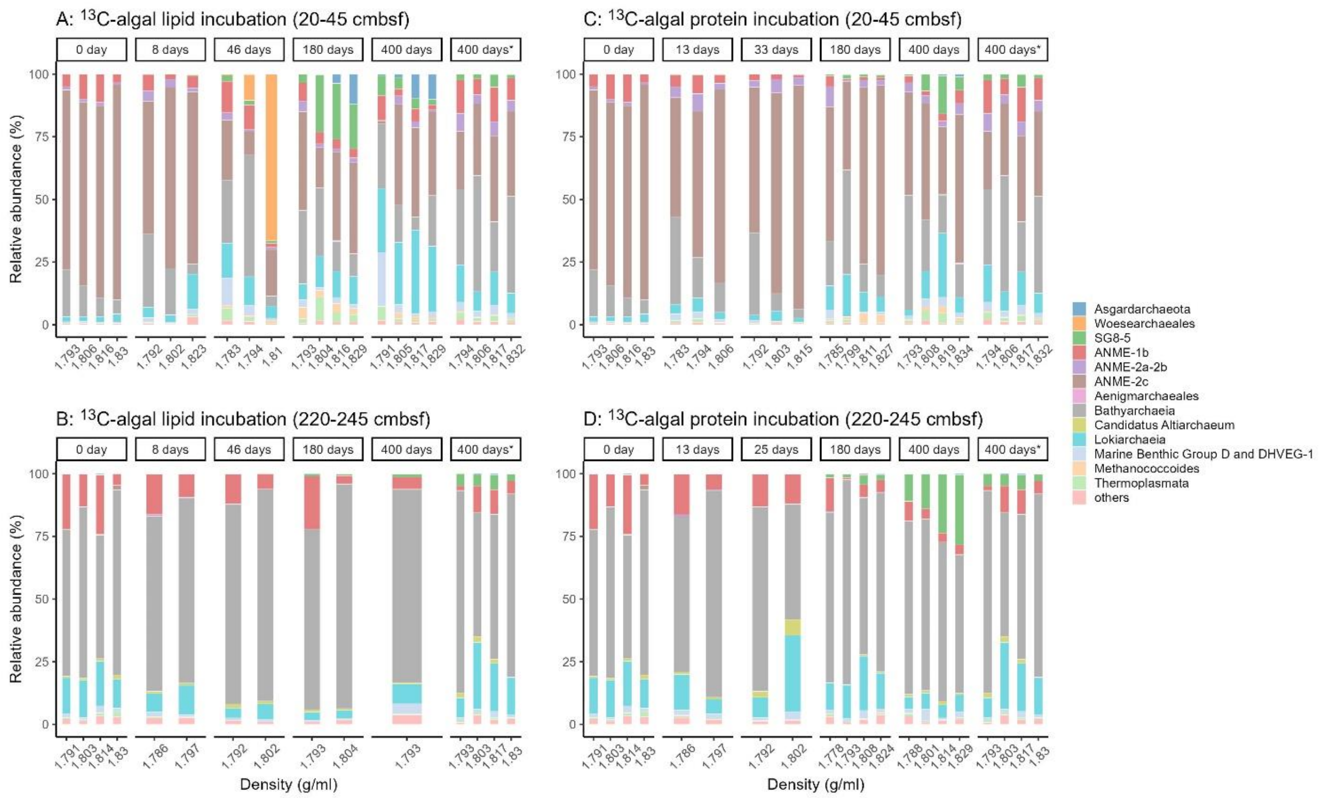

Fig. S8. Archaeal community composition during lipid and protein degradation. RNA density distribution targeting algal lipid and protein archaeal degraders during the ^{13}C-algal lipid and protein incubations. The active archaea are dominated by Asgardarchaeota, Woesearchaeales, and SG8-5. The sequencing data was not retrieved from all experiments due to low RNA recovery.

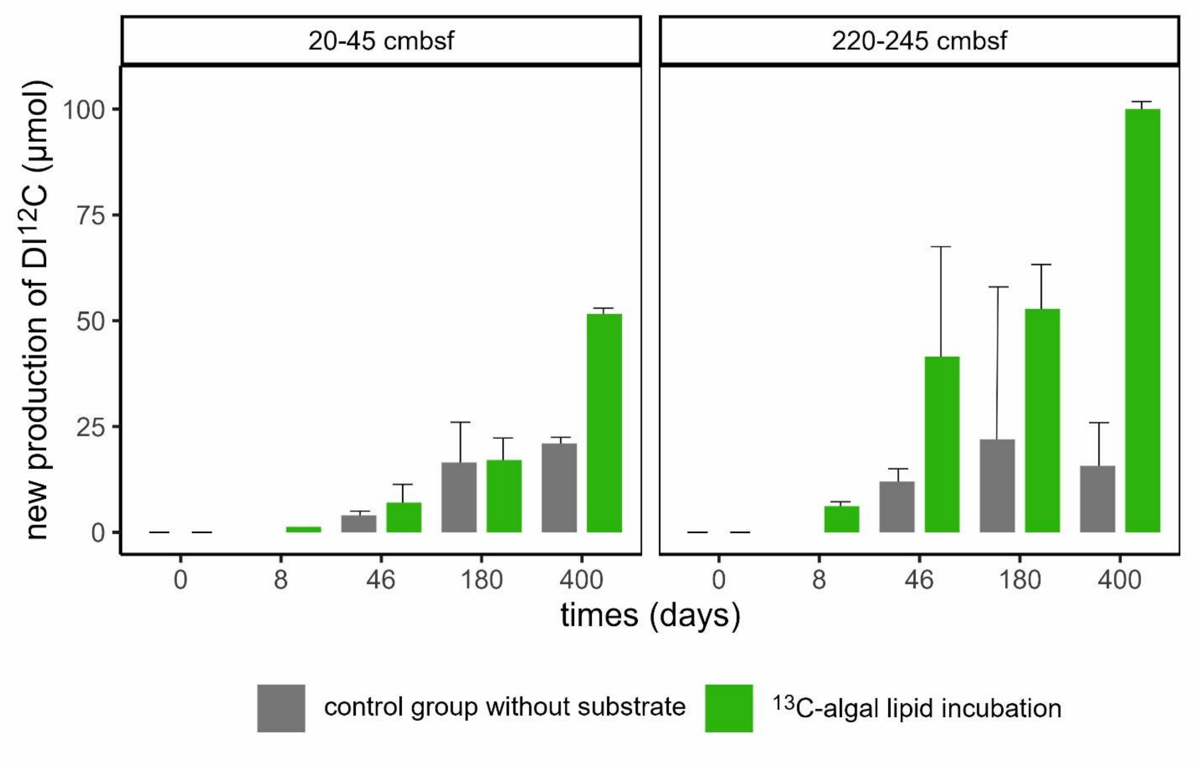

Fig. S9. Extra production of DI^{12}C indicates the priming intensity. The differences in DI^{12}C production between incubations with ^{13}C-algal lipid substrate and the control group without substrate is a clear signal of priming.
